# Substance-related coping behaviours among youth during the early months of the COVID-19 pandemic

**DOI:** 10.1016/j.abrep.2021.100392

**Published:** 2021-11-03

**Authors:** Isabella Romano, Karen A. Patte, Margaret de Groh, Ying Jiang, Terrance J. Wade, Richard E. Bélanger, Scott T. Leatherdale

**Affiliations:** a School of Public Health Sciences, University of Waterloo, Waterloo, ON, Canada; b Applied Research Division, Public Health Agency of Canada, Ottawa, ON, Canada; c Department of Health Sciences, Brock University, St. Catharines, ON, Canada; d VITAM - Centre de recherche en santé durable de l’Université Laval, Québec, QC, Canada; e Department of Pediatrics, Faculty of Medicine, Université Laval, Québec, QC, Canada

**Keywords:** COVID-19 pandemic, Substance use, Coping behaviour, Adolescent health, Mental health

## Abstract

**Objective:**

As impacts of the COVID-19 pandemic continue to unfold, research is needed to understand how school-aged youth are coping with COVID-19-related changes and disruptions to daily life. Among a sample of Canadian youth, our objective was to examine the mental health factors associated with using substances to cope with COVID-19-related changes, taking account of expected sex differences.

**Methods:**

We used online data collected from 7150 students in the COMPASS study, during the early months of the COVID-19 pandemic (May–July 2020) in British Columbia, Ontario, and Quebec, Canada. We specified a sex-stratified, generalized linear mixed model to estimate the likelihood of engagement in substance-related coping behaviours, while testing for the effects of students’ mental health, individual characteristics, and school neighborhood characteristics.

**Results:**

Twelve percent of students (13.4% of females, 9.9% of males) in our sample reported using cannabis, alcohol, cigarettes, and/or vaping to help cope with COVID-19-related changes. Regardless of sex, students with greater depressive symptoms were more likely to engage in substance-related coping (aOR_Female_ = 1.04, 95 %CI[1.01–1.07]; aOR_Male_ = 1.06, 95 %CI[1.013–1.11]). Among females, better psychosocial wellbeing was protective against engagement in substance related-coping (aOR = 0.96, 95 %CI[0.94–0.98]), controlling for current substance use.

**Conclusions:**

Canadian school-aged youth with generally poor mental health may be more likely to have engaged in substance use to help cope with COVID-19-related changes during the first wave of the pandemic, and female youth may be at disproportionate risk of engaging in the behaviour. Ongoing evaluation of the impacts of COVID-19 on youth health is required.

## Highlights

• 12% of youth in our sample used substances to cope with COVID-19-related changes.
• Substance-related coping was more common among females than males in our sample.
• Greater depression was associated with using substances to cope with COVID-19.
• For females, psychosocial wellbeing may be protective of substance-related coping.
• Secondary impacts of COVID-19 pandemic on youth substance use and mental health.

## 1 Introduction

The COVID-19 pandemic has disrupted the lives of school-aged youth across Canada and globally. Since March 12th, 2020, provincial governments have enacted emergency lockdown protocols resulting for most adolescents in shifts to remote learning, cancellation of extra-curricular activities, and closure of recreation- and leisure-based facilities. These measures were taken to help curb community spread and transmission of the novel SARS-CoV-2 coronavirus, although the effectiveness of school closures on transmission control remains unclear (Viner et al., 2020). Given that school is normally a source of structure, routine, and social interaction, there has been an expressed concern over the lasting impact that COVID-19-related school closures and physical distancing measures may have on youth health and wellbeing (Galea et al., 2020, Poletti and Raballo, 2021), especially with respect to the potential of exacerbating existing inequities among youth and their families (Van Lancker & Parolin, 2020).

Some authors have expressed their concerns that the ongoing COVID-19 pandemic and its related lockdown restrictions and limitations be characterised as a traumatic stressor, comparable to other large-scale traumatic events, disasters, or war (Bridgland et al., 2021). Such adverse collective experiences are usually accompanied by population-level increases in mental disorder symptoms (e.g., post-traumatic stress, depression) as well as problematic substance use (Galea et al., 2020, Lee et al., 2007, Neria et al., 2008, Vlahov et al., 2004). Within the context of stress and trauma, substance use has been recognized as playing a role in self-medication (Khantzian, 1997) and shortcut healthier coping (Gezinski et al., 2021, Kuper et al., 2010) processes. Coping skills and behaviours can be framed through a deficit model based on a balance of individuals’ stress and the resources available to them (Cramer et al., 2015, Lazarus and Folkman, 1984). In the presence of excess psychosocial stress, a substantial deficit in one’s healthy coping skills may lead to the development of more maladaptive coping behaviours such as substance use (Britton, 2004).

Trends of increased substance use during the COVID-19 pandemic have been noted among both adults (Canadian Centre on Substance Use and Addiction, 2020, MacMillan et al., 2021, Ornell et al., 2020) and youth (Dumas et al., 2020, Lechner et al., 2020). In a sample of Canadian adolescents, Dumas and colleagues (Dumas et al., 2020) reported that while the total proportion of respondents who reported substance use decreased after COVID-19 physical distancing practices had taken place, those that reported using alcohol and cannabis had done so more frequently. Within a sample of adults in Canada and the U.S., 37.5% reported using alcohol or other substances as a source of coping with the COVID-19 pandemic (MacMillan et al., 2021). In another sample of young adults, more than 40% of participants reported using at least one substance to cope with social conditions of COVID-19 (Cho et al., 2021). Youth have also reported relying on general maladaptive strategies to cope with COVID-19 restrictions (Liang et al., 2020), including cannabis use (Dumas et al., 2020); however, no studies have specifically examined substance-related coping behaviours adopted by youth in Canada during the COVID-19 pandemic. Understanding whether youth may be using substances to cope with COVID-19-related changes can help to inform ongoing harm reduction efforts in the interest of youth health.

Certain factors may increase individuals’ propensity to rely on substance-related coping during the COVID-19 pandemic. Worry and fear specific to COVID-19, for example, have been identified as psychological factors associated with substance use coping behaviours among a small group of adults in the U.S. (Rogers et al., 2020). Individuals with pre-existing mental disorder may be at disproportionate risk for engaging in substance-related coping due to COVID-19 (McKay and Asmundson, 2020, Robillard et al., 2021). Likewise, psychological distress has been associated with increased substance use among youth (Hoffmann, 2016, Sinha, 2008, Wills et al., 2001), whereas strength-based factors such as overall wellbeing and healthy emotion regulation skills can often be protective of youth engagement in substance use and related risks (Butler et al., 2019, Siegel, 2015). Access to substances is also an important factor to consider, especially among child and adolescent populations in comparison to adults.

Coping behaviours are known to differ by sex such that males tend to engage in problem-focused coping while females typically engage in emotion-focused coping, which has been described as less effective (Kelly et al., 2008). Females may also be more likely than males to engage in substance use as a response to stress and negative emotions (Fox & Sinha, 2009). Youth may engage in substance use for a variety of reasons including for social, sensation-seeking, and enhancement or via coping motives (Kuntsche et al., 2005). Substance use motives may also differ by sex; a review showed that while males may often engage in drinking for social and sensation-seeking motives, females are more likely to do so for coping reasons (Kuntsche et al., 2006). However, in general, the prevalence of substance use is typically higher among males (Kuhn, 2015). Despite being disproportionately impacted by COVID-19 (Prowse et al., 2021), some emerging evidence shows that among adults, women may be coping better with COVID-19 than men (Rana et al., 2021). This may not be the case among youth, as other findings have shown that declines in youth mental health during COVID-19 have been more prominent among girls than boys (Magson et al., 2021). Investigation of potential sex- and/or gender-based differences in substance-related coping among youth during COVID-19 is thus warranted.

As the impacts of the COVID-19 pandemic continue to unfold, ongoing research is required to understand how school-aged youth in Canada are coping with COVID-19 and its related changes. Using a school-based sample of Canadian female and male youth, our objectives were threefold: 1) to estimate the prevalence of substance-related coping within the context of the COVID-19 pandemic, 2) to examine the mental health and socio-environmental factors associated with engaging in substance use to cope with COVID-19-related changes, and 3) to determine how these associations may potentially differ across female and male students.

## 2 Methods

### 2.1 Study design

We used data from the COMPASS study – an ongoing (started in 2012–13) prospective cohort study that collects hierarchical data from a rolling sample of Canadian youth and the secondary schools they attend in Alberta, British Columbia, Nunavut, Ontario, and Quebec, Canada (Leatherdale et al., 2014). Each year, full school samples of students are invited to complete a questionnaire pertaining to their behaviour and mental health. COMPASS employs active-information, passive-consent data collection procedures which have been approved by the University of Waterloo Office of Research Ethics and participating school boards. All data collected are anonymously, although a unique identification code is generated by the student to allow data linkage across cycles. Detailed information about COMPASS is available in print (Leatherdale et al., 2014) and online (www.compass.uwaterloo.ca).

COVID-19 was declared a pandemic in March 2020 during COMPASS Year 8 (Y_8_ [2019–20]) data collection. Until this point, yearly COMPASS data collections had been conducted in-person and during class time. As schools in Canada began closing in-person learning due to the enactment of COVID-19 public health measures, the online COMPASS Student Questionnaire (CQ-o) was created and implemented (Reel, Battista, & Leatherdale, 2020). In Canada, 94% of households had access to the internet in 2020 (Statistics Canada, 2021). During school closures, using Qualtrics XM online survey software (Qualtrics, Provo, UT, USA), a link to the CQ-o was emailed to all students by their schools starting May 1, 2020 with the last survey closing on July 6, 2020.

### 2.2 Sample

A total of 9630 students participated in the Y_8_ CQ-o across 51 schools in British Columbia, Ontario, and Quebec (2 in BC, 20 in ON, 29 in QC); data were not collected in Alberta or Nunavut during this period. We deleted participants from our analytic sample if they had missing demographic data (n = 511, 5.3%), and if they self-identified as other than female or male (n = 133, 1.4%) to mitigate issues with small sub-sample cell counts. After cases with missing data in the independent and dependent categories were also removed using listwise deletion (n = 1836, 19.1%), a complete-case analytic sample of 7150 students remained.

The majority of the sample (63%) was female. Mean participant age was 15 years, and 21% identified with a racialized ethnicity. Fewer than one quarter (22%) reported not having any weekly spending money. A majority (61%) of schools were situated within neighborhoods with median household incomes between $50,000 and $75,000. Fewer than 10% of school neighborhoods were considered rural.

### 2.3 Measures

#### 2.3.1 Dependent variable of interest: Substance-related coping behaviours

The CQ-o included the question, “How have you been coping with changes related to COVID-19?” to which students were instructed to mark all that apply from a list of behaviours including the following options: “Using cannabis/marijuana,” “Drinking alcohol,” “Smoking cigarettes,” and “Vaping.” We grouped students according to whether they endorsed a least one of these substance-related coping behaviours (i.e., *yes* to any substance) or not (i.e., *no*, did not engage [*ref*.]). Note that at the time these data were collected, recreational cannabis was legal for adult use in Canada but remained illegal for youth (<19 in BC and ON; <21 in QC) (Parliament of Canada, 2018).

#### 2.3.2 Independent variables of interest: Mental health

**Depressive symptoms.** The Centre for Epidemiological Studies Depression Scale (Revised)-10 item (CESD-R-10) (Andresen et al., 1994, Zhang et al., 2012) was used to assess students’ self-reported symptoms of depression. Using a 4-point Likert scale (0 = “none or<1 day,” 1 = “1–2 days,” 2 = “3–4 days,” 3 = “5–7 days”), students indicated the frequency at which they experienced emotional and psychosomatic symptoms of unipolar depression during the past 7 days. Possible sum scores ranged from 0 to 30, with higher scores indicating greater severity. Internal consistency of the CESD-R-10 in our study sample was high (Cronbach α = 0.94).

**Anxiety symptoms.** The CQ-o also included the Generalized Anxiety Disorder 7-item Scale (GAD-7) (Spitzer et al., 2006). Students reported how often (0 = “not at all,” 1 = several days,” 2 = “over half the days,” 3 = “nearly every day”), if at all, they experienced symptoms of generalized anxiety (e.g., uncontrollable worrying, restlessness, etc.) in the past 2 weeks. Like the CESD-R-10, higher sum scores (0 to 21) on the GAD-7 indicate greater symptom severity. The GAD-7 had high internal consistency in our sample (α = 0.99).

**Psychosocial wellbeing.** A modified version of the 8-item Flourishing Scale (FS) (Diener et al., 2009, Romano et al., 2020) was used as a measure of self-perceived psychosocial wellbeing. Using a 5-point Likert scale (1 = “strongly disagree,” 2 = “agree,” 3 = “neither agree nor disagree,” to 4 = “agree,” 5 = “strongly agree”), students indicated their level of agreement with a series of statements related to positive mental health and functioning (e.g., “I am engaged and interested in my daily activities”). Possible sum scores ranged from 8 to 40 and higher scores indicated greater wellbeing, or flourishing. Internal consistency of the FS was high (α = 0.99).

**Emotion regulation skills.** Items from the Difficulties in Emotion Regulation Scale (DERS) (Neumann et al., 2010) were used to assess students’ emotional intelligence and regulation problems. Based on previous validation studies, the CQ-o included the highest loading item from each of the 6 DERS subscales (Patte et al., 2017). Students indicated how often (1 = “almost never,” 2 = “sometimes,” 3 = “about half the time,” 4 = “most of the time,” 5 = “almost always”) each statement (e.g., “when I’m upset, I lose control over my behaviour,” “when I’m upset, I have difficulty concentrating”) applied to them. Higher scores (ranging from 6 to 30) indicated greater dysregulation. The DERS items had high internal consistency (α = 0.99).

#### 2.3.3 Substance use

General measures of substance use were also included in the CQ-o, which we used to describe our study sample. Students were asked how often they engaged in cannabis use and binge-drinking (defined as 5 or more drinks on one occasion) in the past 12 months, and we categorized frequencies of at least once per month as current use. Frequency of cigarette use and vaping were asked within the past 30 days, and we defined use of at least one day as current. Students were also asked how often they drank alcohol or used cannabis when they were by themselves in the last 30 days. We defined engagement in solitary substance use as any solitary use of alcohol or cannabis at least once in the past 30 days.

#### 2.3.4 Sociodemographic characteristics

Students self-reported on their sex/gender with the question, “are you female or male?” using the following response options: “female,” male,” “I describe my gender in a different way,” and “I prefer not to say.” For analytic purposes, we relied on students who indicated female and male sex. Age (in years) was also collected in the CQ-o. We also included race/ethnicity and weekly spending money as relevant sociodemographic characteristics in our analyses, given their association with both substance use and mental health in existing youth literature. To capture race/ethnicity, students were asked to describe themselves by selecting one or more of the following categories: Asian, Black, Indigenous (First Nations/Métis/Inuit), Latin American/Hispanic, white, or other. We re-categorized students according to whether or not they indicated a racialized (i.e., Asian, Black, Indigenous, Latin American/Hispanic, other, mixed/multiple) or non-racialized (i.e., white) ethnic identity. As a proxy measure for socioeconomic status (SES) and part-time employment, students also reported their weekly available spending money (zero, $1–20, $21–100, $100+, don’t know).

#### 2.3.5 School neighborhood characteristics

To assess whether participants’ substance-related coping might be affected by environmental contexts of their school neighbourhood, we also included measures of median income and urbanicity which may influence substance use more generally. School neighborhood median income was identified through the 2016 Statistics Canada Census Profile using postal codes (Statistics Canada, 2016). Median income was categorized into 3 groups (< $50,000, $50,000–$75,000, >$75,000). School urbanicity data were obtained using GeoSearch lookup on city name (Statistics Canada, 2017). Rural areas were defined by populations <1000 or by population density <400/km^2^.

### 2.4 Analyses

First, we computed descriptive statistics (*χ^2^*, *t*) to compare student characteristics, mental health, substance use, and substance-related coping by student sex (female, male). We reported the prevalence of current substance use (cannabis, binge-drinking, cigarettes, vaping) and compared it with the prevalence at which students used each to cope with COVID-19-related changes. We also reported the prevalence of solitary substance use and compared it with engagement in substance-related coping. Next, a sex-stratified, generalized linear mixed model (GLMM) was used to estimate the likelihood of engagement in substance-related coping behaviours among female and male students. The GLMM approach was chosen to account for the hierarchical structure of the data.

The model tested for the effects of students’ mental health (CESD-R-10, GAD-7, FS, and DERS scores), individual characteristics (age, race/ethnicity, weekly available spending money), and school neighborhood characteristics (median income, urbanicity), while adjusting for province and school clustering. We specified a logit link function to account for our binary dependent variable (substance coping, *yes* vs. *no*). Unadjusted and adjusted odds ratios (ORs) are reported with 95% confidence intervals. The unadjusted ORs represent the effects of each variable in the GLMM before controlling for other factors, while the adjusted ORs (Model I and II) represent the independent effects of each variable with all variables included (i.e., full model). First, in Model I, we did not control for students’ current substance use; Model II includes substance use. We tested for and did not detect multicollinearity between independent variables in our adjusted models. We used SAS v9.4 statistical software (SAS Institute, 2016).

### 2.5 Missing data

Missing values were mainly observed in measures of substance-related coping (16.7%) and mental health (CESD-R-10, 18.67%; GAD-7, 16.2%; FS, 15.6%; DERS, 16.2%); few students were missing data for sex (1.6%), age (1.6%), race/ethnicity (1.8%), or weekly spending money (4.7%). We identified a pattern of monotone missingness as a probable result of questionnaire drop-off (i.e., partial completion of the CQ-o). Missing data analyses are presented in Supplementary File A, in which we used logistic regression models to estimate students’ likelihood of missingness for measures of coping and mental health. For all measures modelled, students were generally more likely to have missing data if they were male or identified with a racialized ethnicity.

## 3 Results

### 3.1 Prevalence of substance-related coping

Overall, 12% (n = 863) of students in our sample reported engaging in any substance use to help cope with COVID-19-related changes between May and July 2020, including 13% (n = 602) of female students and 10% (n = 261) of male students (*χ*^2^ = 21.3, *p* < 0.0001; Table 1). As shown in Table 1, students most commonly reported using alcohol to cope (8%), followed by vaping (6%), cannabis (4%), and cigarettes (1%). There were significant sex differences in the prevalence of coping with more females reporting coping with COVID-19 by using cannabis (*χ*^2^ = 5.8, *p* < 0.05), alcohol (*χ*^2^ = 12.6, *p* < 0.001), and vaping (*χ*^2^ = 11.3, *p* < 0.001). Use of cigarettes to cope with COVID-19-related changes did not differ among females and males. Most students who engaged in substance-related coping were exclusively using one substance (7.4% of total sample) rather than multiple (4.7% of total sample) to cope (*χ*^2^ = 21.5, *p* < 0.0001).

**Table 1:** Descriptive comparisons by sex among COMPASS Y_8_ students (May-July 2020), N = 7150.

		Sex, *n* (%)		*χ^2^*, *t*	*p*
Measure	**Full sample**	**Females**	**Males**		
Age, years					
Mean age (SD)	15.1 (1.5)	15.1 (1.5)	14.9 (1.5)	**−5.1**	**<0.0001**
Race/ethnicity					
Non-racialized	5617 (78.6)	3453 (77.1)	2164 (81.0)	**14.9**	**0.0001**
Racialized	1533 (21.4)	1025 (22.9)	508 (19.0)		
Weekly spending money					
Zero	1571 (22.0)	853 (19.1)	718 (26.9)	**80.6**	**<0.0001**
$1–20	1396 (19.5)	851 (19.0)	545 (20.4)		
$21–100	1187 (16.6)	791 (17.7)	396 (14.8)		
$101+	1298 (18.2)	822 (18.4)	476 (17.8)		
Don’t know	1698 (23.7)	1161 (25.9)	537 (20.1)		

CESD-R-10					
Mean score (SD)	8.8 (6.1)	9.9 (6.3)	7.1 (5.3)	**−20.4**	**<0.0001**
GAD-7					
Mean score (SD)	6.1 (5.5)	7.2 (5.7)	4.3 (4.6)	**−23.2**	**<0.0001**
FS					
Mean score (SD)	32.8 (5.5)	32.6 (5.5)	33.1 (5.4)	**4.1**	**<0.0001**
DERS items					
Mean score (SD)	14.3 (4.7)	14.9 (4.8)	13.4 (4.2)	**−14.5**	**<0.0001**

Current (past month) substance use^1^					
No	5723 (80.2)	3507 (78.4)	2216 (83.1)	**22.7**	**<0.0001**
Yes, any substance	1415 (19.8)	964 (21.6)	451 (16.9)		
Cannabis	440 (6.2)	295 (6.6)	145 (5.4)	**3.9**	**0.0482**
Binge-drinking	814 (11.4)	542 (12.1)	272 (10.2)	**6.1**	**0.0133**
Cigarettes	243 (3.4)	163 (3.6)	80 (3.0)	2.1	0.1446
Vaping	928 (13.0)	658 (14.7)	270 (10.1)	**31.0**	**<0.0001**
Solitary use, alcohol or cannabis					
No	641 (46.2)	422 (44.5)	219 (49.9)	**7.1**	**0.0077**
Yes	747 (53.8)	527 (55.5)	220 (50.1)	0.1	0.7535

Substance-related coping with COVID-19					
No	6287 (87.9)	3876 (86.6)	2411 (90.2)	**21.3**	**<0.0001**
Yes, any substance	863 (12.1)	602 (13.4)	261 (9.9)		
Type of substance used to cope^1^					
Cannabis	255 (3.6)	178 (4.0)	77 (2.9)	**5.8**	**0.0159**
Alcohol	565 (7.9)	393 (8.8)	172 (6.4)	**12.6**	**0.0004**
Cigarettes	77 (1.1)	43 (1.0)	34 (1.3)	1.5	0.2159
Vaping	453 (6.3)	318 (7.1)	135 (5.1)	**11.8**	**0.0006**
Number of substances used to cope					
Used one substance	527 (7.4)	371 (8.3)	156 (5.8)	**21.5**	**<0.0001**
Used multiple substances	336 (4.7)	231 (5.2)	105 (3.9)		

**Total:**	7150 (100.0)	4478 (62.6)	2672 (37.4)		

*Note.* SD = standard deviation. CESD-R-10 = Centre for Epidemiological Studies Depression Scale (Revised)-10. GAD-7 = Generalized Anxiety Disorder 7-item scale. FS = Flourishing Scale. DERS = Difficulties in Emotion Regulation Scale.

1 Substance categories are not mutually exclusive.

**Fig. 1:**
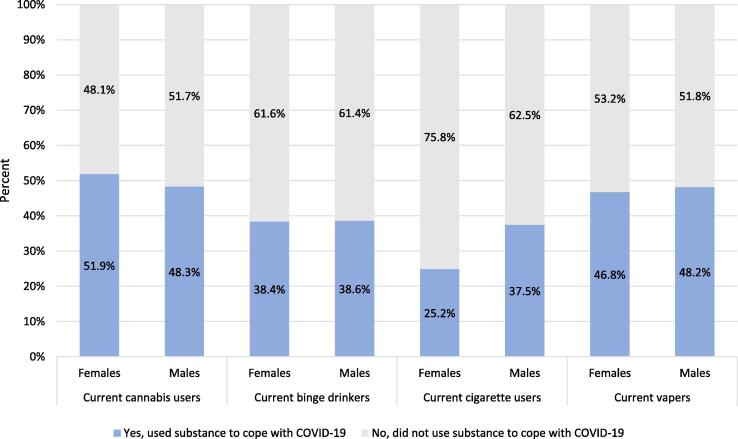
Prevalence of substance-related coping with COVID-19 among current substance users in COMPASS Y_8_ (May–July 2020), N = 7150. Note. Fig. 1 shows the prevalence of substance-related coping with COVID-19 among female and male students who reported current use of cannabis, binge-drinking, cigarettes, and vaping. For example, among students who reported current cannabis use, 51.9% of females and 48.3% of males engaged in cannabis use to help cope with COVID-19-related changes.

Within this same sample, 20% (n = 1415) reported current substance use in general (6% cannabis, 11% binge-drinking, 3% cigarettes, 13% vaping) and of these, 54% reported solitary use. Rates of current substance use and engagement in solitary substance use are shown in Table 1 by sex. As shown in Fig. 1, we also reported the prevalence of substance-related coping among females and males who used each substance generally. Among students who engaged in any solitary use of alcohol or cannabis in the past 30 days, fewer than half (46%) reported using substances to cope with COVID-19-related changes and these rates did not significantly differ by sex (46% female, 47% male; *χ^2^ =* 0.1, *p =* 0.7676).

### 3.2 Sex-stratified GLMM results

Table 2 presents results of the sex-stratified GLMM models estimating the log-odds of engaging in substance use to cope with COVID-19-related changes. In the model showing unadjusted estimates, all measures of mental health were significantly associated with substance-related coping. Increases in sum scores along the CESD-R-10, GAD-7, and DERS scales were associated with incremental increases in the log-odds of substance-related coping; likewise, increasing FS scores were associated with lower log-odds of engaging in substance-related coping. These unadjusted effects were present among both females and males.

**Table 2:** Sex-stratified generalized linear mixed model results predicting engagement in substance-related coping behaviours (any substance) during the early months of the COVID-19 pandemic (May-July 2020).

		OR (95% CI)				
Measure	**Females**			**Males**		
	**Unadjusted**	**Model I**	**Model II**	**Unadjusted**	**Model I**	**Model II**
Age, years	1.45 (1.36, 1.55)***	1.35 (1.26, 1.45)***	1.18 (1.08, 1.30)***	1.75 (1.58, 1.93)***	1.57 (1.41, 1.75)***	1.37 (1.19, 1.58)***
Race/ethnicity						
Non-racialized (*ref.*)	1.00	1.00	1.00	1.00	1.00	1.00
Racialized	0.88 (0.70, 1.12)	0.83 (0.65, 1.07)	1.04 (0.77, 1.41)	1.03 (0.72, 1.48)	1.99 (0.67, 1.44)	0.98 (0.61, 1.60)
Weekly spending money						
Zero (*ref.*)	1.00	1.00	1.00	1.00	1.00	1.00
$1–20	1.29 (0.82, 1.82)	1.51 (1.06, 2.16)*	1.47 (0.95, 2.30)	1.02 (0.62, 1.69)	1.16 (0.69, 1.95)	0.84 (0.45, 1.58)
$21–100	2.28 (1.65, 3.13)***	2.42 (1.72, 3.39)***	1.69 (1.10, 2.60)*	2.61 (1.67, 4.07)***	2.22 (1.39, 3.54)***	1.37 (0.76, 2.48)
$101+	3.68 (2.72, 4.98)***	3.06 (2.21, 4.24)***	1.62 (1.07, 2.45)*	4.74 (3.17, 7.08)***	3.31 (2.15, 5.10)***	1.18 (0.67, 2.08)
Don’t know	1.41 (1.03, 1.94)*	1.53 (1.09, 2.14)*	1.47 (0.97, 2.21)	1.43 (0.90, 2.28)	1.42 (0.87, 2.29)	1.11 (0.62, 2.01)

School neighborhood median income						
$25,000–50,000 (*ref.*)	1.00	1.00	1.00	1.00	1.00	1.00
$50,000–75,000	0.99 (0.65, 1.52)	1.14 (0.75, 1.72)	1.19 (0.85, 1.68)	0.99 (0.56, 1.78)	1.10 (0.63, 1.91)	1.13 (0.71, 2.10)
$75,001+	0.65 (0.38, 1.09)	0.86 (0.52, 1.43)	1.08 (0.71, 1.64)	0.52 (0.25, 1.08)	0.66 (0.33, 1.33)	0.77 (0.36, 1.66)
School neighborhood urbanicity						
Rural (*ref.*)	1.00	1.00	1.00	1.00	1.00	1.00
Urban	1.05 (0.58, 1.88)	1.28 (0.72, 2.27)	1.48 (0.95, 2.30)	1.06 (0.50, 2.24)	1.22 (0.57, 2.62)	1.07 (0.47, 2.44)

CESD-R-10						
Estimate (SE)	1.09 (1.08, 1.11)***	1.05 (1.03, 1.07)***	1.04 (1.01, 1.07)*	1.09 (1.07, 1.12)***	1.06 (1.03, 1.11)**	1.06 (1.01, 1.11)*
GAD-7						
Estimate (SE)	1.09 (1.08, 1.11)***	1.01 (0.99, 1.04)	1.01 (0.98, 1.04)	1.08 (1.06, 1.11)***	1.01 (0.97, 1.05)	0.99 (0.94, 1.05)
FS						
Estimate (SE)	0.92 (0.90, 0.93)***	0.96 (0.94, 0.98)***	0.95 (0.93, 0.98)***	0.95 (0.93, 0.98)***	1.00 (0.97, 1.03)	1.02 (0.98, 1.06)
DERS items						
Estimate (SE)	1.11 (1.09, 1.13)***	1.04 (1.02, 1.07)**	1.01 (0.98, 1.04)	1.09 (1.06, 1.12)***	1.04 (0.99, 1.08)	1.02 (0.97, 1.07)

*Note.* Models estimate the log-odds of any engagement in substance-related coping, compared to none. Unadjusted ORs refer to the bivariate effects of each independent variable in the model. Model I shows the full adjusted model, without substance use included. Model II shows the full adjusted model, controlling also for current substance use. All ORs control for province and school clustering.

*ref.* = reference category. OR = odds ratio. CI = confidence interval. SE = standard error. CESD-R-10 = Centre for Epidemiological Studies Depression Scale (Revised)-10. GAD-7 = Generalized Anxiety Disorder 7-item scale. FS = Flourishing Scale. DERS = Difficulties in Emotion Regulation Scale. * *p* < 0.05, ** *p* < 0.01, *** *p* < 0.001

Among females, results from the adjusted Model I (not including current substance use) showed that greater depressive symptoms and difficulties in emotion regulation were associated with a higher likelihood of using substances to cope with COVID-19-related changes. Greater overall psychosocial wellbeing among females was negatively associated with substance-related coping behaviour. Depressive symptoms were the only significant mental health effect among males in the adjusted Model I.

In Model II, the effects of depressive symptoms persisted among females and males after further adjusting for current substance use. For every unit increase in CESD-R-10 sum score, there was a 4% and 6% increase in the log-odds of engaging in substance-related coping behaviours among females and males, respectively, while controlling for current substance use. For female students in adjusted Model II, we found there were lower log-odds of substance-related coping with increases in psychosocial wellbeing; every unit increase along the FS was associated with a 5% decrease in the log-odds of substance-related coping.

For both female and male students in our sample, higher adolescent age was associated with significantly greater log-odds of engaging in substance-related coping behaviours. Log-odds were also higher among female and male students reporting greater weekly spending money, compared to those without any available spending money. The effects of age and weekly spending money persisted after full adjustment for current substance in Model II among females, and only for age among males. School neighborhood median income and urbanicity did not have significant effects on substance-related coping.

## 4 Discussion

Using self-reported data collected online from Canadian youth during the early months of the COVID-19 pandemic (May-July 2020), this study aimed to investigate student engagement in substance use as a method of coping with COVID-19-related changes. We identified that approximately 12% of secondary school students in our sample reported substance-related coping using at least one of alcohol, cannabis, cigarettes, or vaping which also accounted for between 25 and 50% of students who used substances more generally. While the prevalence of substance-related coping in our youth sample is lower than what was recently observed within adult samples (Cho et al., 2021, MacMillan et al., 2021), it remains a concern that a significant portion of youth in Canada were relying on substance use as a strategy for coping with changes brought forth by the pandemic, particularly given the added substance accessibility challenges youth may have face during the early months of the lockdown, compared to adults. We also found that youth with generally poorer mental health – particularly those experiencing greater levels of depressive symptoms – were more likely to engage in substance-related coping. Several child health advocates have expressed their concern that the pandemic may disproportionately impact or exacerbate problems among youth with existing mental health concerns (Fegert et al., 2020). As the current pandemic continues to bring change and uncertainty to the lives of school-aged youth in Canada, understanding the impact that COVID-19-related restrictions and physical distancing measures on youth should be a priority for research and ongoing program and policy decision-making.

Compared to males, more female students in our sample reported engaging in substance use to cope with COVID-19-related changes. This finding is consistent with evidence that females may be more likely to use substances as a coping strategy (Fox & Sinha, 2009). Moreover, among female students in our sample, those reporting greater levels of overall psychosocial wellbeing were less likely to engage in substance-related coping – even after controlling for current substance use. Positive mental health factors should be noted as protective against maladaptive coping in response to COVID-19-related changes, during a time where other common and more adaptive youth coping assets (e.g., social encountering, sports participation) were restricted. Based on our findings, this may be especially important for female youth, who in a recent study reported disproportionately greater changes in their subjective wellbeing due to COVID-19 (Pigaiani et al., 2020). Existing evidence shows that Canadian youth who are flourishing are less likely to engage in higher-risk behaviours such as substance use (Butler et al., 2019, Holligan et al., 2020, Romano et al., 2019). Efforts to mitigate the risks of COVID-19-related changes for school-aged youth may consider adopting strength-based approaches (Yamaguchi et al., 2020) to enhance student resiliency and adaptive coping skills.

We found that, for both female and male students, higher depressive symptoms were associated with higher likelihood of engaging in substance-related coping. These effects persisted even after we adjusted for students’ current substance use. Symptoms of depression, such as anhedonia, have been identified as predictors of youth cannabis and nicotine use (Leventhal et al., 2017, Stone et al., 2017), and adolescents experiencing mood disorders may be likely to adopt self-medicating behaviours using alcohol and other substances to cope with their symptoms (Audrain-Mcgovern et al., 2009, Turner et al., 2018). In Canada, Cost and colleagues (Cost et al., 2021) recently found that a portion of children and adolescents with psychiatric diagnoses have experienced deterioration in their symptoms, namely as a function of increased stress from COVID-19-related social isolation (Loades et al., 2020). This is consistent with research that found daily routines provided by school and extra-curricular activities to be protective of youths’ mental and physical health (Brazendale et al., 2017, Bridley and Jordan, 2012), while unstructured time has been identified as a risk factor for substance use behaviours among youth (Mahoney & Stattin, 2000).

Controlling for students’ socio-environmental characteristics also shows that other factors may be associated with maladaptive coping strategies among youth. For both female and male students, increased age was a significant correlate of substance-related coping during COVID-19. As children and adolescents mature toward young adulthood, emotion regulation skills become increasingly sophisticated (Theurel & Gentaz, 2018), and this developmental period is important for promoting adaptive coping skills in response to stressors (Horn et al., 2011). Students in our sample were also more likely to report engaging in substance-related coping if they had greater weekly spending money. This is consistent with observations in existing youth substance use literature, where more spending money has been associated with higher rates of substance use (Czoli et al., 2015, Gaete and Araya, 2017, Markowitz and Tauras, 2006, Romano et al., 2019, Shi et al., 2018, Zuckermann et al., 2020)—likely by way of increased accessibility. It may be reasonable to assume that the same pattern could extend to youths’ substance-related coping behaviours, although further research is required. We note also that since this finding did not persist among male students after accounting for current substance use, accessibility may play a unique role in females’ engagement in substance-related coping.

Emotion dysregulation was associated with increased substance-related coping for females, but not while accounting for current substance use. From a developmental perspective, this effect may have been apparent among females as they tend to perceive stressors as more severe (Pigaiani et al., 2020) – and are likely to express a wider variety of complex emotions (Bailen et al., 2019) – than males. However, as this emotion dysregulation effect did not persist after controlling for current substance use, additional research is needed.

Interestingly, we noted significant differences among females and males in the prevalence of using cannabis, alcohol, and vaping to cope with COVID-19-related changes, in which a larger proportion of females reported engaging in each substance-specific behaviour than males. This is inconsistent with established evidence that substance use may be more common among males than females in general (Kuhn, 2015), but consistent with evidence that females may be predisposed to engaging in substance-related coping than males (Fox & Sinha, 2009). Engagement in substance use as a means of self-medication and coping with negative affect is thought to contribute to an increased risk of developing substance use disorders among females compared to males (Becker et al., 2012). The same was not found for cigarette use; but despite a narrowing gap over the past two decades, existing evidence shows that rates of cigarette use among males remains higher than among females in Canada (Reid et al., 2019). Observed sex and gender gaps in cannabis use, drinking, and vaping are typically not as prominent (Health Canada, 2019). In our current study, all substances were collapsed into a single indicator of substance-related coping; as more data become available, future research may benefit from looking at specific substances in detail.

The COMPASS system is continuing to collect these data during the 2020–21 school year, and it will be important to extend our analyses longitudinally to further assess trends in coping behaviours while changes due to COVID-19 have normalized. Research efforts should also consider social substance use behaviours (e.g., social gathering, sharing vaping devices) on COVID-19 transmission among youth. Dumas and colleagues noted that some Canadian adolescents reported using substances with peers virtually, and others have continued to use substances with their peers in person despite COVID-19 physical distancing measures (Dumas et al., 2020). We speculate that a subset of adolescents in our sample continued to use substances with peers in-person, placing them at increased risk of COVID-19 transmission; however, this cannot be verified with COMPASS data. Continued use despite public health restrictions may also be indicative of dependence among youth, particularly with respect to cigarettes and nicotine-containing vapes; further research is required. More than 50% students who used substances in our sample had engaged in solitary use of either alcohol or cannabis in the past 30 days, and substance-related coping was commonly reported in nearly half of solitary users. Solitary substance use is a uniquely risky behaviour which may pose further risk for poor psychosocial and behavioural outcomes (Tucker et al., 2006). Additional research is needed to assess the impact of COVID-19 on solitary substance use behaviour and its potential mental health implications.

### 4.1 Strengths and limitations

Using timely data collected through the COMPASS system, this study is relevant to understanding the impact that the ongoing COVID-19 pandemic and related changes may have on the behavioural and mental health of youth in Canada. COMPASS was able to swiftly adapt its procedures to enable ongoing data collection despite school closures. These large-scale data collection systems such as COMPASS will be key for facilitating natural experimental evaluations which can inform evidence-based decision-making in real time (Leatherdale, 2019). This study is also strengthened by a robust sample size and use of multi-level mixed modelling to account for the hierarchical and clustered structure of the data.

Limitations should be noted. First, data were collected via purposive sampling and are not nationally-representative – findings do not necessarily generalize to other school-aged youth in Canada. Second, these data do not capture usual rates of substance-related coping or motivations among youth, and so we cannot discern whether substance-related coping changed among youth as a function of COVID-19. Beyond possibly using substances to cope with COVID-19-related changes, these data do not provide further information about youth motives to use substances during the pandemic despite public health restrictions. Third, substance-related coping may be under-reported due to social desirability bias or perceived consequences since the legal federal age permitting use of cannabis, alcohol, cigarettes, and vapes is 18 in Canada (with the exception of 19 in British Columbia and Ontario, 21 for cannabis in Quebec). To mitigate this risk, COMPASS uses an active-information, passive-consent data collection procedure that promotes honest reporting (Thompson-Haile et al., 2013) as all data are collected anonymously using a unique self-generated identification code. Third, another possible source of confounding may be bias due to self-selection. Rather than completing the paper-based CQ at school alongside their classmates, student participants agreed to complete the CQ-o on their own at home and may represent a unique subset of individuals (Schaurer & Weiß, 2020). Selection bias may be relevant in explaining the differences we found between adjusted GLMM model estimates among females and males, as female students were more likely to participate in the COMPASS CQ-o altogether. As such, these data may not adequately represent eligible male students.

As noted, respondents who did not self-identify as female or male were excluded from our analyses due to a small subsample size (n = 155). This precludes us from making valid inferences about non-binary or non-*cis*-sexual/*cis*-gendered youth, who may be particularly vulnerable to the psychosocial impacts of COVID-19-related changes (Fish et al., 2020). This limitation should be addressed in future research using large samples and comprehensive measures of both sex and gender identity. We also note that students in our sample were less likely to have complete data if they were male or racialized – this should be recognized as a possible source of bias. Lastly, our findings should be interpreted with the caveat that data were collected during the early months of the pandemic, which has continued to impact Canadian youth to different degrees over time. Ongoing longitudinal research is needed and COMPASS data collections for the 2020–2021 school year are underway.

## 5 Conclusion

Our current study sought to assess substance-related coping behaviours among female and male high school-aged youth during the early months of the COVID-19 pandemic in Canada. In summary, our findings indicate that a portion of youth reported engaging in use of cannabis, alcohol, cigarettes, or vaping to help cope with COVID-19-related changes, and that greater depressive symptoms were associated with increased risk of substance-related coping among both females and males. We also found that substance-related coping may be more common among females – among whom psychosocial wellbeing was protective – although, more research is needed to confirm possible sex-based differences. This emphasizes the need for ongoing monitoring and evaluation of the secondary impacts that the COVID-19 pandemic and its effect on youths’ mental and behavioural health.

## Funding sources

The COMPASS study has been supported by a bridge grant from the CIHR Institute of Nutrition, Metabolism and Diabetes (INMD) through the “Obesity – Interventions to Prevent or Treat” priority funding awards (OOP-110788; awarded to SL), an operating grant from the CIHR Institute of Population and Public Health (IPPH) (MOP-114875; awarded to SL), a CIHR project grant (PJT-148562; awarded to SL), a CIHR bridge grant (PJT-149092; awarded to KP/SL), a CIHR project grant (PJT-159693; awarded to KP), and by a research funding arrangement with Health Canada (#1617-HQ-000012; contract awarded to SL), a CIHR-Canadian Centre on Substance Abuse (CCSA) team grant (OF7 B1-PCPEGT 410-10-9633; awarded to SL), and a SickKids Foundation New Investigator Grant, in partnership with CIHR Institute of Human Development, Child and Youth Health (IHDCYH) (Grant No. NI21-1193; awarded to KAP) funds a mixed methods study examining the impact of the COVID-19 pandemic on youth mental health, leveraging COMPASS study data. The COMPASS-Quebec project additionally benefits from funding from the Ministère de la Santé et des Services sociaux of the province of Québec, and the Direction régionale de santé publique du CIUSSS de la Capitale-Nationale.

## CRediT authorship contribution statement

**Isabella Romano:** Conceptualization, Methodology, Formal analysis, Writing - original draft. **Karen A. Patte:** Supervision, Data curation, Funding acquisition, Resources, Writing - review & editing. **Margaret de Groh:** Methodology, Resources, Writing - review & editing. **Ying Jiang:** Methodology, Resources, Writing - review & editing. **Terrance J. Wade:** Writing - review & editing. **Richard E. Bélanger:** Data curation, Writing - review & editing. **Scott T. Leatherdale:** Supervision, Data curation, Funding acquisition, Resources, Writing - review & editing.

## Declaration of Competing Interest

The authors declare that they have no known competing financial interests or personal relationships that could have appeared to influence the work reported in this paper.
