# Supplementary material for: Substance-related coping behaviours among youth during the early months of the COVID-19 pandemic
Source: Addict Behav Rep. 2021 Nov 3;14:100392. doi: 10.1016/j.abrep.2021.100392 (PMC8565916; doi:10.1016/j.abrep.2021.100392)
Supplement: Supplementary Data 1 [file mmc1.docx]

| **Supplementary File A**. *Logistic regression models estimating the log-odds of missing data for measures of substance-related coping, depression, and anxiety among COMPASS Y_8_ students (May-July 2020)* | | | | | |
| --- | --- | --- | --- | --- | --- |
|  | aOR (95% CI) | | | | |
| Measure | **Model I** | **Model II** | **Model III** | **Model IV** | **Model V** |
| Sex |  |  |  |  |  |
| Female (*ref.*) | 1.00 | 1.00 | 1.00 | 1.00 | 1.00 |
| Male | **1.33 (1.17, 1.51)***** | **1.24 (1.10, 1.40)***** | **1.26 (1.11, 1.44)***** | **1.23 (1.08, 1.41)**** | **1.34 (1.18, 1.52)***** |
| Age, years |  |  |  |  |  |
| Estimate (SE) | 1.02 (0.98, 1.07) | 0.98 (0.94, 1.03) | 1.01 (0.96, 1.06) | 1.02 (0.97, 1.07) | 1.02 (0.97, 1.07) |
| Race/ethnicity |  |  |  |  |  |
| Non-racialized (*ref.*) | 1.00 | 1.00 | 1.00 | 1.00 | 1.00 |
| Racialized | **1.34 (1.15, 1.56)***** | 1.43 (1.24, 1.64)*** | **1.27 (1.08, 1.48)**** | **1.36 (1.16, 1.59)***** | **1.40 (1.20, 1.64)***** |
| Weekly spending money |  |  |  |  |  |
| Zero (*ref.*) | 1.00 | 1.00 | 1.00 | 1.00 | 1.00 |
| $1-20 | 1.05 (0.86, 1.28) | 1.06 (0.88, 1.27) | 0.99 (0.81, 1.21) | 1.04 (0.85, 1.27) | 1.03 (0.84, 1.27) |
| $21-100 | 1.06 (0.86, 1.31) | 1.03 (0.84, 1.25) | 0.95 (0.76, 1.17) | 0.94 (0.75, 1.17) | 1.02 (0.82, 1.27) |
| $101+ | 1.21 (0.98, 1.49) | 1.04 (0.85, 1.27) | 1.02 (0.82, 1.26) | 1.01 (0.81, 1.26) | 1.11 (0.90, 1.28) |
| Don’t know | 1.19 (0.99, 1.43) | 1.19 (0.99, 1.41) | 1.18 (0.98, 1.42) | 1.20 (0.99, 1.45) | **1.30 (1.08, 1.57)*** |
| *Note.*  Model I estimates the log-odds of missing data for engagement in substance-related coping; Model II estimates the log-odds of missing data in CESD-R-10 score; Model III estimates the log-odds of missing data in GAD-7 score; Model IV estimates the log-odds of missing data in FS score; Model V estimates the log-odds of missing data in DERS items score. For each model outcome, *missing* = 1 vs. *not missing* = 0. All models are adjusted for province. *ref.* = reference category. aOR = adjusted odds ratio. CI = confidence interval. SE = standard error.  * *p* < 0.05, ** *p* < 0.01, *** *p* < 0.001 | | | | | |
